# Inhibition of Influenza H7 Hemagglutinin-Mediated Entry

**DOI:** 10.1371/journal.pone.0076363

**Published:** 2013-10-23

**Authors:** Aleksandar Antanasijevic, Han Cheng, Duncan J. Wardrop, Lijun Rong, Michael Caffrey

**Affiliations:** 1 Department of Biochemistry & Molecular Genetics, University of Illinois at Chicago, Chicago, Illinois, United States of America; 2 Department of Microbiology & Immunology, University of Illinois at Chicago, Chicago, Illinois, United States of America; 3 Department of Chemistry, University of Illinois at Chicago, Chicago, Illinois, United States of America; University of Nebraska – Lincoln, United States of America

## Abstract

The recent outbreak of H7N9 influenza in China is of high concern to public health. H7 hemagglutinin (HA) plays a critical role in influenza entry and thus HA presents an attractive target for antivirals. Previous studies have suggested that the small molecule *tert*-butyl hydroquinone (TBHQ) inhibits the entry of influenza H3 HA by binding to the stem loop of HA and stabilizing the neutral pH conformation of HA, thereby disrupting the membrane fusion step. Based on amino acid sequence, structure and immunogenicity, H7 is a related Group 2 HA. In this work we show, using a pseudovirus entry assay, that TBHQ inhibits H7 HA-mediated entry, as well as H3 HA-mediated entry, with an IC50∼6 µM. Using NMR, we show that TBHQ binds to the H7 stem loop region. STD NMR experiments indicate that the aromatic ring of TBHQ makes extensive contact with the H7 HA surface. Limited proteolysis experiments indicate that TBHQ inhibits influenza entry by stabilizing the H7 HA neutral pH conformation. Together, this work suggests that the stem loop region of H7 HA is an attractive target for therapeutic intervention and that TBHQ, which is a widely used food preservative, is a promising lead compound.

## Introduction

The membrane glycoproteins hemagglutinin (HA) and neuraminidase (NA) play critical roles in influenza infection [1]. Antigenic properties are used to classify HA and NA of influenza strains into subtypes (HA: H1-17 and NA: N1-9) with some strains posing enormous threats to human health. For example, the pandemic H1N1 influenza outbreak of 1918 resulted in over 50 million deaths worldwide with a fatality rate of 3% and, despite improved vaccination efforts and better treatments, seasonal influenza is still responsible for greater than 250,000 deaths per year worldwide [2], [3]. Of very high concern is the recent outbreak of H7N9 influenza in China, which has exhibited a mortality rate of >20% [4], [5], [6]. Current treatments for influenza include Tamiflu (oseltamivir) and Relenza (zanamivir), which target NA, and Symmetrel (amantadine) and Flumadine (rimantidine), which target the M2 channel [7]. Unfortunately, resistance is increasing in circulating influenza strains. For example, the 2008–2009 H1N1 strain exhibited ∼100% resistance against Tamiflu [8]. Moreover, the recent H7N9 strain contains a sequence rendering it insensitive to the M2 channel blockers [9] and some H7N9 strains are also showing resistance to Tamiflu and Relenza [10]. As a consequence, novel antiviral treatments against new targets are highly desirable.

HA, as well as the analogous envelope proteins from Ebola, HIV, and SARS-CoV, mediates virus entry through receptor binding and conformational changes that result in fusion of the viral and target cell membranes [11], [12], [13]. Based on sequence, structure and immunogenicity, the HA fall into 2 phylogenetic groups [14], [15], [16]. Examples of Group 1 HA are those of H1 and H5; examples of Group 2 HA are those of H3 and H7. In all cases, HA is synthesized as a precursor (HA0), which is subsequently cleaved to form a non-covalent complex consisting of HA1, the receptor binding subunit, and HA2, the subunit that mediates membrane fusion [1]. During the entry process, HA undergoes a series of binding and pH-induced conformational changes that result in binding of the virus to the plasma membrane, entry of the virus into the endosome, exposure of the HA fusion peptide fusion, fusion of the viral and endosomal membranes, and finally release of the viral RNA into the cytoplasm [1]. The critical nature of HA function makes it an attractive target for therapeutics (small molecules, proteins or antibodies) designed to inhibit entry at either the binding or fusion steps (i.e. binding or fusion inhibitors, [7], [17], [18], [19], [20], [21], [22], [23], [24], [25], [26], [27]. In the case of the fusion inhibitors, they are thought to often bind to the stem loop region of HA2 and act by stabilizing the pre-fusion (neutral pH) conformation of HA [23], [25], [26], [27]. For example, the small aromatic molecule *tert*-butyl hydroquinone (TBHQ) inhibits H3 HA-mediated influenza entry by binding to the stem loop of HA [17], [23], [28]. TBHQ is a widely used antioxidant food preservative that is approved to concentrations up to 600 mM [29], [30]. Interestingly, the fusion inhibitors, including TBHQ, appear to act in a group specific manner [23], [25], [26], [27] (i.e. inhibitors of Group 1 HA, such as H5, do not inhibit Group 2 HA, such as H7). In this work we compare the inhibitory and binding properties of TBHQ to H7 HA with that of H3 HA. Together, our work suggests that the stem loop region of H7 HA is an attractive target for therapeutic intervention and that TBHQ is a promising lead compound.

## Experimental Procedures

### Materials

HA from subtypes H7 A/Netherlands/219/2003 and H3 A/Brisbane/10/2007 were obtained from BEI Resources (Manassas, VA). The recombinant HAs are full length, prepared in cell culture, and glycosylated. The purity of the HAs were verified by SDS-PAGE. TBHQ was obtained from Sigma (St. Louis, MO) and monoclonal antibody F49 was obtained from Takara Bio (Mountain View, CA).

### Viral Entry Assays

The inhibitory properties of TBHQ were tested in a pseudovirus entry assay as previously described by our groups [31], [32], [33], [34]. Briefly, plasmids pHA-H7 or pHA-H3 (bearing the HA for strains A/Netherlands/219/2003 and A/Aichi/2/1968, respectively), pNA (bearing influenza neuraminidase) and pNL4-3.Luc.R-E- were co-transfected by PEI (PolySciences, Inc., Warrington, PA) into 293T cells, which were maintained in Dulbecco's medium with 10% FBS, and 1% penicillin-streptomycin. Forty-eight hours post-transfection, the medium was harvested and filtered through a 0.45 micron filter to make the virus stock. For assay of viral entry, A549 cells, which were maintained in Dulbecco's medium with 10% FBS supplemented with 1% penicillin-streptomycin, were seeded to 2×10^4^ cells/well of a 24 well cell culture plate in a volume of 0.5 mL. The following day, 500 µL of the virus stock was added to each of the wells of the A549 cells after removal of the medium. In experiments with TBHQ, the pseudovirions were premixed with the appropriate amount of TBHQ from a DMSO stock solution (dilutions of 1∶200). The plates were incubated at 37°C in a CO_2_ incubator. After approximately 3–5 hours, the virus was aspirated and replaced with A549 medium and the cells were allowed to rest for another 40 hours. Luciferase activity was measured using the Luciferase Assay System from Promega (Madison, WI) and a Berthold FB12 luminometer running Sirius software. The experiments were run in triplicate from transfection to assay of luciferase activity and thus the uncertainties represent all stages of the experiment. In all cases, the viral entry levels fell within the linear range of detection (i.e. the values of the wild-type and mutants never exceeded 3×10^6^ Relative Light Units, [31]). Cytotoxicity was determined using the CellTiter-Glo kit (Promega).

### NMR Experiments

NMR experiments were performed on a Bruker 900 MHz AVANCE spectrometer equipped with a cryogenic triple resonance probe. Experimental conditions were 0.5 µM HA and 50–100 µM TBHQ in 20 mM PO_4_/pH 7.4, 100 mM NaCl in 90% ^1^H_2_O, 10% ^2^H_2_O (WaterLOGSY) or 100% ^2^H_2_O (STD) at 25°C in 3 mm NMR tubes. The WaterLOGSY experiments were performed as previously described [35], [36]. Water was selectively saturated using a 2 msec square shaped pulse with a mixing time of 2 sec and a relaxation delay of 2.5 sec. STD experiments were performed as previously described [37], [38]. In these experiments, protein ^1^H were saturated with a train of 50 msec gaussian-shaped pulses at 100 Hz power for 1 sec with “on” resonance saturation at −1 ppm and “off” resonance saturation at 30 ppm (the relaxation delay was 2.5 sec before the saturating pulses). Spectra were processed by NMRPipe with a 5 Hz line broadening function and analyzed by NMRDraw [39]. Competition WaterLOGSY experiments were performed with a 2∶1 ratio of F49 monoclonal antibody to HA (the spectra in the presence of antibody have been corrected for any binding of TBHQ to F49). Relative % STD was defined as 100 X STD_obs_/STD_max_ where STD  = ΔI/I_off_, ΔI = I_off_–I_on_ and I_off_ and I_on_ are the intensities observed for the various resonances after the “off” and “on” presaturation of HA. Errors in the WaterLOGSY and STD were estimated as ΔI/I_ref_((N_ΔI_/ΔI)^2^+ (N_Ioff_/I_ref_)^2^)^0.5^ [38], where NΔI and N_Iref_ are the noise calculated by NMRDraw in the appropriate spectrum (no antibody in WaterLOGSY and I_off_ in the STD).

### HA Stability Assays

In the limited proteolysis experiments, ∼10 µM H7 HA was pre-incubated in the presence of ∼330 µM TBHQ for 15 min at 37°C (control experiments were performed in 0.7% DMSO). Phosphate-Citrate buffer stock solutions at the appropriate pH were added to result in final buffer and TBHQ concentrations of 50 mM and 250 μM, respectively. The buffered solution was then incubated for 30 min at 37°C. Next, the solutions were neutralized to pH 8.0 and incubated with 2 µg of trypsin for 30 min at 37°C. The proteolysis was stopped by adding 11 µL of 5X SDS running buffer and heating the samples at 95°C for 10 min. After SDS PAGE and Coomassie staining, the HA bands were quantified using Bio-Rad Image Lab 2.0.1. Subsequently, the HA band intensities were fit to % Native  = 100/(1+ (I_obs_/[H+]_mp_)^n^), where I_obs_ is the observed intensity at a particular pH, [H^+^]_mp_ is the midpoint [H+] concentration, and n =  Hill Coefficient, using Kaleidagraph 4.1.3.

## Results and Discussion

### TBHQ Inhibits the Entry of H3 and H7 HA

**Figure 1:**
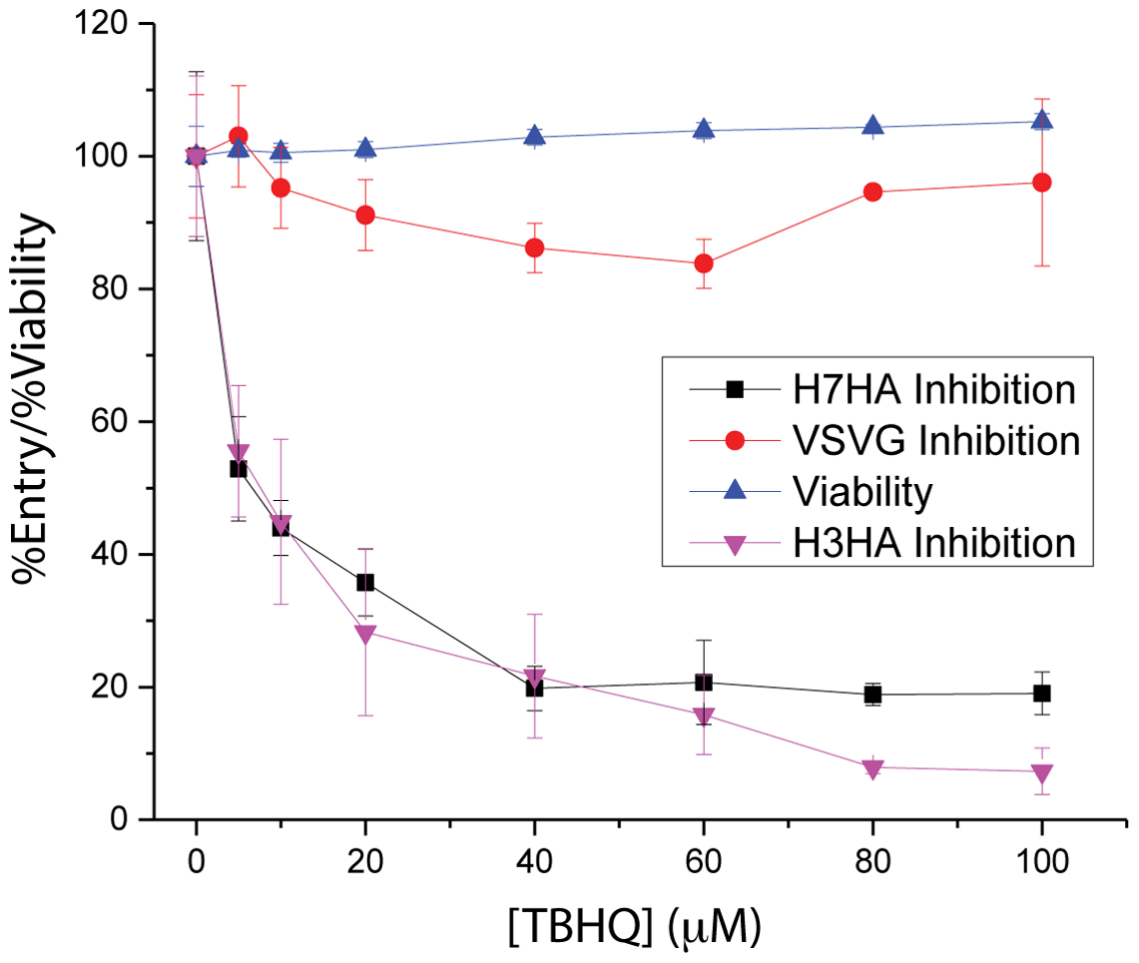
TBHQ inhibits H3 and H7 HA-mediated entry. Each concentration point was performed in triplicate.

Previously, our groups have shown that pseudovirus containing envelope proteins from Ebola, HIV, Influenza and SARS-CoV on a background of HIV core proteins are useful surrogates for infectious virus [31], [32], [33], [34]. As noted above, the small molecule TBHQ has been reported to inhibit H3 HA-mediated influenza entry [17], [28], a Group 2 HA that is phylogenetically related to H7 HA. Accordingly, we first tested the inhibitory properties of TBHQ using pseudovirions containing H3 HA. As shown by Figure 1, TBHQ inhibits H3 HA-mediated entry with an IC50∼7 µM (purple triangles), which is in agreement with the previously reported IC50 for inhibition of H3 influenza infection [17], [28]. Next, we tested the inhibitory properties of TBHQ using pseudovirions containing H7 HA. As shown by Figure 1, TBHQ inhibits H7 HA-mediated entry with an IC50∼6 µM (black squares). In a next step, specificity to HA is shown by the lack of detectable inhibition for VSVG-mediated entry, pseudovirion containing an unrelated envelope protein (Figure 1, red circles, [31], [32], [33], [34]. Finally, the toxicity of TBHQ was determined to be CC50>100 µM as shown in Figure 1 (blue triangles). Taken together, these experiments show that the small molecule TBHQ inhibits H7 HA-mediated entry with a similar potency to that of influenza H3 infection.

### TBHQ Binds to the Stem Loops of H3 and H7 HA

**Figure 2:**
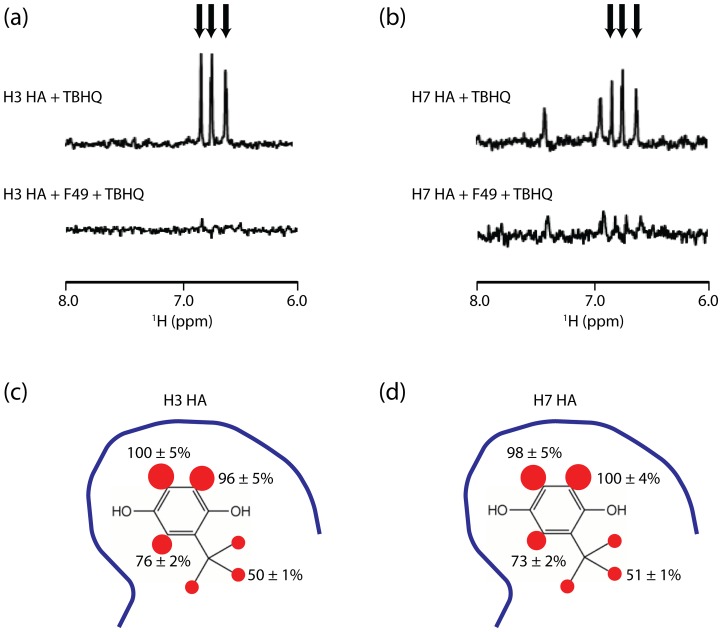
TBHQ binds to the H3 and H7 HA stem loops. (a) WaterLOGSY NMR of TBHQ binding to H3 HA in the presence and absence of monoclonal antibody F49. (b) WaterLOGSY NMR of TBHQ binding to H7 HA in the presence and absence of monoclonal antibody F49. In (a) and (b) the aromatic resonances of TBHQ are denoted by arrows. (c) Relative STD signals of TBHQ in the presence of H3 HA. (d) Relative STD signals of TBHQ in the presence of H7 HA. The blue line represents the protein surface. The size of the red spheres represent the magnitude of the observed STD for each ^1^H.

WaterLOGSY NMR has previously been shown to be useful for the detection of small molecule interactions with large protein complexes [35], including the ∼200 kDa trimer of influenza HA [36]. Accordingly, we first tested whether TBHQ binds to recombinant H3 HA using the WaterLOGSY experiment. As shown in Figure 2a, the presence of positively phased TBHQ resonances in the NMR experiment clearly indicates that TBHQ binds to H3 HA. Previously, TBHQ was shown to bind to the stem loop of H3 HA by x-ray crystallography [23]. Moreover, the monoclonal antibody F49 has been reported to bind to the stem loop of H3 HA [40]. Consequently, we tested TBHQ binding to the H3 HA stem loop using a WaterLOGSY-based competition assay in the presence of antibody F49. As shown in Figure 2a, the addition of monoclonal antibody F49 significantly decreases the observed binding of TBHQ (the average reduction of TBHQ resonance intensities was 79% +/−2%), suggesting that the antibody is displacing the compound. Next, we characterized the binding of TBHQ to H7 HA. As shown in Figure 2b, TBHQ binds to H7 HA and, based on the competition NMR experiment in the presence of antibody F49, TBHQ binds to the stem loop region of HA (the average reduction of TBHQ resonance intensities was 69% +/−3%). We further characterized the TBHQ interaction with H3 and H7 HA using the STD NMR experiment, which gives insight into the small molecule ^1^H in closest contact with the protein surface [37], [38]. A summary of the relative intensities of the TBHQ STD spectrum is shown in Figures 2c and 2d, for the HA of H3 and H7, respectively. Based on this analysis, the TBHQ contacts to the H3 and H7 HA surfaces are similar with the aromatic ^1^H in closest contact. Interestingly, in both cases the *tert*-butyl methyl ^1^H exhibit significantly less STD intensity, suggesting that they are more distant from the protein surface and thus they may present attractive sites for modifications designed to increase affinity. Taken together, the NMR experiments indicate that TBHQ binds to the stem loop of H3 and H7 HA with a similar mode of binding.

### TBHQ Stabilizes the Neutral pH Conformation of H3 and H7 HA

**Figure 3:**
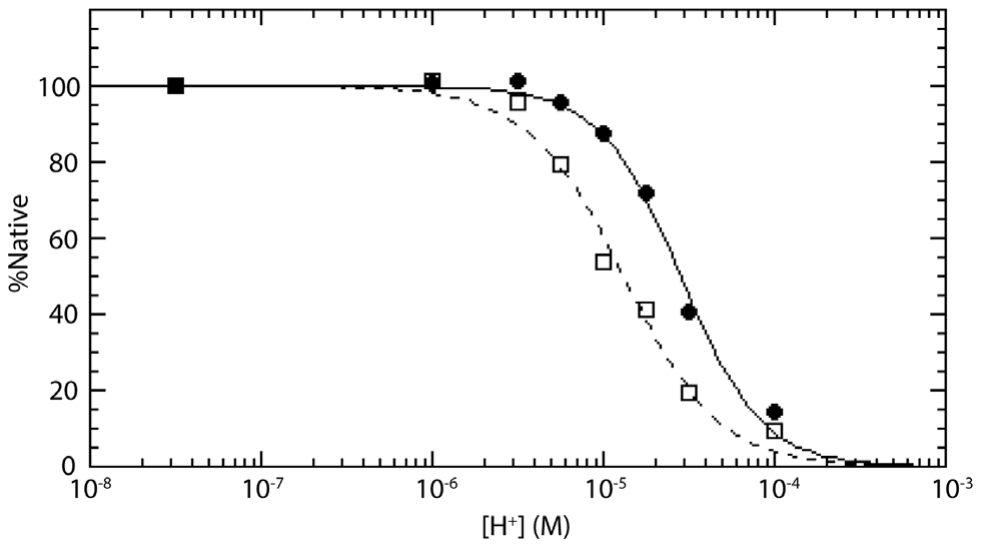
TBHQ stabilizes the neutral pH conformation of H7 HA. Limited proteolysis experiment for H7 HA in the presence (open squares, dotted line) and absence (filled circles, solid line) of TBHQ at different pH. The curves correspond to fits using [H^+^]_mp_  = 1.30×10^−5^ and n = 1.6 in the absence of TBHQ and [H+]_mp_  = 2.83×10^−5^ and n = 1.8 in the presence of TBHQ (c.f. Experimental Procedures).

During influenza entry, the low pH of the endosome triggers a large, irreversible conformational change in HA, which is necessary for membrane fusion [1]. Previously, TBHQ has been shown to inhibit H3 HA-mediated entry by stabilization of the HA neutral pH conformation using a limited proteolysis assay, in which the low pH conformation is more sensitive to proteolysis [17]. Accordingly, we used a limited proteolysis assay to test the stability of H7 HA at different pH in the presence and absence of TBHQ. As shown in Figure 3, H7 HA becomes more susceptible to proteolysis at a midpoint corresponding to pH ∼4.9 (filled circles, solid line), which is similar to that previously described for a cell-cell fusion assay of H7 HA [41]. Interestingly, the addition of TBHQ appears to stabilize H7 HA to proteolysis by shifting the midpoint to pH ∼4.6 (open squares, dotted line). We note that a similar sized shift to lower pH has been observed for an unrelated fusion inhibitor of H1 HA influenza using a cell-based assay [42]. In summary, the limited proteolysis assays suggest that TBHQ inhibits H7 HA-mediated entry by stabilizing the neutral pH conformation and thereby disallows the conformational change necessary for membrane fusion.

## Conclusions

Our work shows for the first time that TBHQ inhibits H7 HA-mediated entry in a similar manner to that of the previously characterized H3 HA. Moreover, our studies suggest that TBHQ binds to the stem loop of H7 HA and that it acts by stabilizing the neutral pH conformation, thereby delaying conformational changes necessary for fusion of the virus and endosome membranes. Taken together, we suggest that TBHQ is an attractive lead compound for the development of antivirals directed at H7 HA. Notably, the sequence identity between the H7 HA strain used in the present studies and that responsible the new H7N9 outbreak in China is >96% with 100% sequence identity in the stem loop region. Nonetheless, TBHQ exhibits relatively modest potency and thus it is not suitable as therapeutic agent without further development. However, we note that our STD NMR experiments suggest sites for modification of TBHQ. On the other hand, TBHQ is a widely used food preservative [29], [30] and thus it may present the potential for addition to animal feeds, including those for bird, the transmission agent of H7N9 influenza [9], as an antiviral agent. Presently, there are no small molecule FDA-approved therapeutics targeting HA. Consequently, HA entry inhibitors, including those to H7 HA, present unique opportunities for treating influenza, particularly for drug-resistant influenza strains, for which resistance is for therapeutics targeting the NA and/or the M2 channel and not HA.
